# Tocilizumab and Thromboembolism in COVID-19: A Retrospective Hospital-Based Cohort Analysis

**DOI:** 10.7759/cureus.15208

**Published:** 2021-05-24

**Authors:** Kok Hoe Chan, Bhavik Patel, Bishnu Podel, Maria E Szablea, Hamid S Shaaban, Gunwant Guron, Jihad Slim

**Affiliations:** 1 Internal Medicine, Saint Michael's Medical Center, Newark, USA; 2 Medical Education, Saint Michael's Medical Center, Newark, USA; 3 Infectious Diseases, Saint Michael's Medical Center, Newark, USA; 4 Hematology and Oncology, Saint Michael's Medical Center, Newark, USA

**Keywords:** coronavirus 2019, sars-cov-2, tocilizumab, il-6, hypercoagulable state, thrombosis, il-6 receptor antagonist

## Abstract

Background

Tocilizumab, an interleukin-6 (IL-6) receptor antagonist, has been used in patients with coronavirus disease 2019 (COVID-19) as an anti-cytokine agent. IL-6 also plays a complex role in hemostasis and thrombosis. We observed a transient elevation of D-dimer in our patients who received tocilizumab, which triggered this study.

Methods

A retrospective hospital-based cohort analysis of patients with confirmed COVID-19 who received tocilizumab during the study period of March 15, 2020, to May 20, 2020, was conducted. We retrieved demographic, clinical, and laboratory data, and patients who were receiving therapeutic anticoagulation therapy prior to tocilizumab administration were excluded. Descriptive analysis was performed, and the cause of death and trends of D-dimer and inflammatory markers were studied.

Results

Out of the 436 confirmed COVID-19 patients admitted during the study period, 24 met the inclusion criteria. Their median age was 47.5 years. They were 18 males and 6 females; 15 patients survived and nine expired. Of the group that survived, 12 received therapeutic anticoagulation. Of the seven patients who did not receive therapeutic anticoagulation, four expired (one from sepsis and three probably from thromboembolic complications) compared to five deaths in the 17 patients who received therapeutic anticoagulation (four from sepsis and one possibly from thromboembolic complications).

Conclusions

The interplay between IL-6, IL-6 receptor antagonist, and venous thromboembolism is complex. We observed a transient elevation of D-dimer in COVID-19 patients who received tocilizumab, and a trend toward increased death secondary to thromboembolism. This observation is novel and highlights the potential thrombophilic side effects of tocilizumab.

## Introduction

Coronavirus disease 2019 (COVID-19) is a major public health emergency. COVID-19 has multifaceted presentations, and while the majority of patients are asymptomatic or present with mild disease, there is a subgroup of patients who tend to present with severe disease. Cytokine storm mediated by proinflammatory cytokines such as interleukin-6 (IL-6) and tumor necrosis factor-alpha (TNF-α) is probably the culprit for severe COVID-19 [1]. Exuberant inflammatory response with complications related to cytokine release syndrome have been observed and proposed as leading to critical and fatal illness [2,3].

Currently, there are two recombinant humanized monoclonal antibodies targeting the IL-6 receptor that are approved by the FDA in the USA: sirukumab and tocilizumab. There are multiple case reports and observational studies reporting the use of tocilizumab in patients with COVID-19 from around the world [4-6]. In the observational study by Xu et al., 21 patients with severe or critical COVID-19 infections showed rapid fever reduction and reduced oxygen requirement after receiving tocilizumab [4], highlighting the potential role of IL-6 in modulating the disease activity.

To the best of our knowledge, tocilizumab has not been shown to increase the risk of thrombosis or hypercoagulable state. Nonetheless, we have observed an elevation of D-dimer in the patients receiving tocilizumab while the rest of the inflammatory markers (ferritin, lactate dehydrogenase [LDH], and C-reactive protein [CRP]) were down-trending, with an increased mortality related to thromboembolism, raising the possibility that tocilizumab may be promoting a prothrombic state. In this retrospective hospital-based cohort study, we report the clinical outcomes and dissect the potential interplay between tocilizumab and thrombosis.

This article was previously posted to the Research Square preprint server on July 16, 2020.

## Materials and methods

A retrospective analysis was conducted of patients admitted to our hospital between March 15, 2020, and May 20, 2020. Inclusion criteria were as follows: (1) positive SARS-CoV-2 RT-PCR (severe acute respiratory syndrome coronavirus 2 reverse transcription polymerase chain reaction) and (2) received tocilizumab as part of their COVID-19 regimen. The only exclusion criterion was prior anticoagulation use.

**Figure 1:**
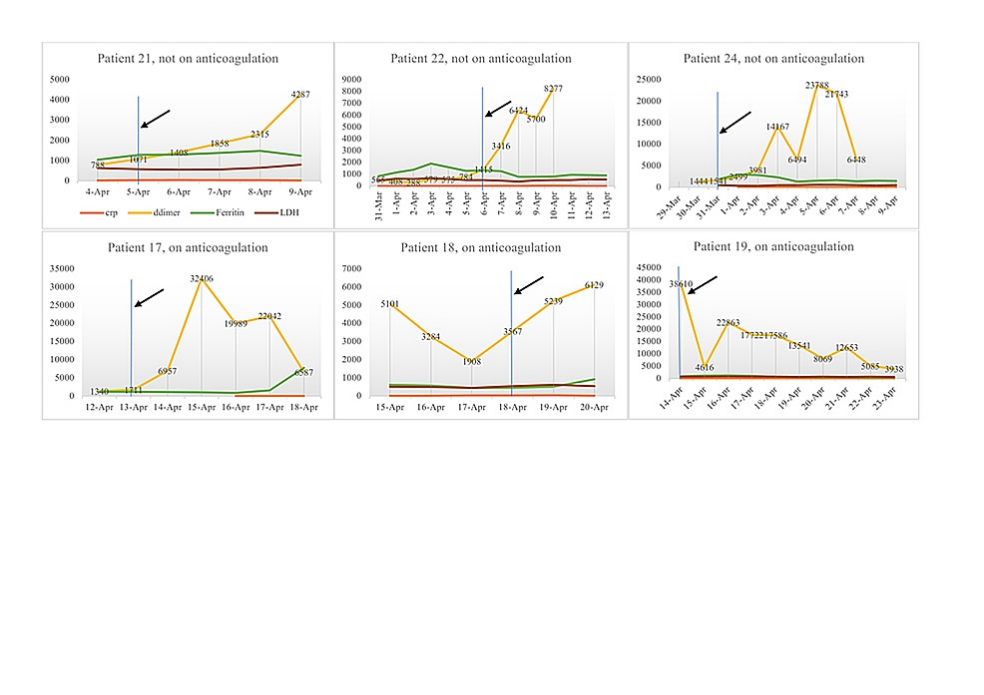
Trend of D-dimers and inflammatory markers (CRP, LDH, and ferritin) of the patients who expired. Blue line and black arrow indicate the time of the tocilizumab given, orange line indicates CRP, yellow line indicates D-dimer, green line indicates ferritin, and brown line indicates LDH. CRP, C-reactive protein; LDH, lactate dehydrogenase

Demographic analysis was performed, and data were expressed as counts, percentages, or median. Death was determined as thromboembolic disease if the patient expired with increasing D-dimer, while ferritin and CRP were decreasing and as septic shock if cultures were positive, increasing vasopressor requirement and/or increasing CRP and/or ferritin. Death was unlikely due to thromboembolic if D-dimer was <1,500 at the time of death. The trends of D-dimer and inflammatory markers of the patients who did not receive anticoagulation are plotted and shown in Figure 1. A waiver of HIPAA (Health Insurance Portability and Accountability Act) privacy authorization was been obtained through the hospital Local Institutional Review board.

## Results

Between March 15, 2020, and May 20, 2020, 24 SARS-CoV-2 RT-PCR tested positive patients received one dose of tocilizumab 400 mg IV. Patients’ demographics and outcomes are tabulated in Table 1.

**Table 1:** Demographic, clinical outcomes, and cause of death after tocilizumab. AA, African American; AHRF, acute hypoxic respiratory failure; CRP, C-reactive protein

No.	Age, years	Gender	Race	No. of days of symptoms	No. of days from symptoms to tocilizumab	Therapeutic anticoagulation	Outcome	Cause of death
1	38	Male	Hispanic	7	11	Yes	Survived	-
2	41	Male	Hispanic	3	3	Yes	Survived	-
3	43	Female	Hispanic	9	11	Yes	Survived	-
4	44	Male	Other	10	10	Yes	Survived	-
5	44	Male	Hispanic	7	11	Yes	Survived	-
6	46	Male	Hispanic	7	9	Yes	Survived	-
7	50	Male	Other	14	19	Yes	Survived	-
8	57	Male	Hispanic	14	16	Yes	Survived	-
9	60	Female	AA	7	10	Yes	Survived	-
10	60	Female	Hispanic	7	10	Yes	Survived	-
11	61	Female	Other	14	15	Yes	Survived	-
12	67	Male	AA	5	10	Yes	Survived	-
13	34	Male	Hispanic	7	10	No	Survived	-
14	44	Female	Other	3	3	No	Survived	-
15	60	Female	Hispanic	7	7	No	Survived	-
16	37	Male	Hispanic	2	3	Yes	Expired	Septic shock with acute rise of CRP/ferritin
17	42	Male	Hispanic	5	9	Yes	Expired	AHRF likely due to thromboembolic event
18	49	Male	Hispanic	7	8	Yes	Expired	Septic shock and multiorgan failure increasing pressor requirement (three pressors)
19	52	Male	Hispanic	7	9	Yes	Expired	Septic shock (blood culture positive for *Neisseria sicca*)
20	62	Male	Hispanic	7	14	Yes	Expired	Septic shock with increasing CRP/ferritin
21	35	Male	Hispanic	14	18	No	Expired	AHRF likely due to thromboembolic event
22	39	Male	Hispanic	7	8	No	Expired	AHRF likely due to thromboembolic event
23	52	Male	Hispanic	5	7	No	Expired	Septic shock and severe *Clostridiodes difficile*
24	60	Male	Hispanic	7	10	No	Expired	AHRF likely due to thromboembolic event

Their median age was 47.5 years (range: 32-67 years). They were 18 males and 6 females. As for ethnicity, 17 (74%) were Hispanic, two (9%) were African American, and four (17%) belonged to other ethnicities. Of the 24 patients, 15 (65%) patients survived and nine (35%) patients expired. Therapeutic anticoagulation with either low molecular weight heparin or unfractionated heparin was given to 17 (71%) patients. Of the 15 patients who survived, 12 (80%) patients received therapeutic anticoagulation. Only five (56%) patients in the expired group received therapeutic anticoagulation. Four patients who were not anticoagulated expired (one from sepsis and three probably from thromboembolic complications) compared to five deaths in the 17 patients who received therapeutic anticoagulation (four from sepsis and one possibly from thromboembolic complications).

Out of 24 patients, 18 (75%) had elevation of D-dimer after tocilizumab administration, while all the inflammatory markers (ferritin, LDH, and CRP) were trending down. Moreover, the elevation was more pronounced in the group of patients who expired and not on therapeutic anticoagulation (Figure 1).

## Discussion

The association between tocilizumab and thrombosis, to our knowledge, has never been reported. We observed an elevation of D-dimer after tocilizumab administration. Moreover, in four patients who were not anticoagulated and expired, there was a profound elevation of D-dimer, and three of them expired probably secondary to thromboembolic events. As compared to those who were anticoagulated, most of them expired due to sepsis and only one expired likely secondary to thromboembolic event.

The interplay between IL-6, IL-6 receptor antagonist, and venous thromboembolism is complex. IL-6 is a proinflammatory cytokine that is involved in inflammation, autoantibody production, endothelial regeneration, and permeability, as well as hematopoiesis [7]. IL-6 is produced by a variety of cells including T-lymphocytes, monocytes, endothelial cells, and fibroblasts [7]. Stone et al. has demonstrated an increased risk of deep vein thrombosis with tumor-derived IL-6 in a mouse model of ovarian cancer via the induction of hepatic thrombopoietin, promoting thrombus formation [8]. Moreover, IL- 6 has been reported to contribute to deep vein thrombosis by dysregulation of miR-338-5p expression [9].

On the contrary, Nosaka et al. has recently published a research article highlighting the role of IL-6 in thrombus resolution, in which suppression of IL-6 may, in fact, result in the growth of the thrombus, as demonstrated in the rat model [10]. Moreover, IL-6 antagonist has been associated with a reduced level of factor XIII, chimerin, and plasminogen activator inhibitor [11]. The pivotal role of factor XIII is in fibrin stabilization, thus the decrease of factor XIII as a result of IL-6 antagonist will lead to thrombus instability, which may also contribute to this thrombophilic state.

Thrombosis is a complex phenomenon, and the hemostasis balance between thrombus formation rate and resolution is important. IL-6 in this context seems to play a complex role in both thrombus formation and resolution. This assumption has been strengthened by the observations that the administration of tocilizumab, an IL-6 antagonist, transiently elevated the D-dimer, which is a marker of coagulability, though more studies are needed to understand the pathophysiological role of IL-6 in hemostasis and thrombosis. Our observation suggests that it may be beneficial to use therapeutic anticoagulation in COVID 19 patients receiving tocilizumab, as they already are at high risk of thrombosis.

## Conclusions

The interplay between IL-6, IL-6 receptor antagonist, and venous thromboembolism is complex. We observed a transient elevation of D-dimer in COVID-19 patients who received tocilizumab. This observation is novel and highlights the potential thrombophilic side effects of tocilizumab. Prospective randomized controlled trials will need to measure this effect in order to come up with a firm clinical recommendation in the future.
